# A pilot study on the genetic diversity of *Mycobacterium tuberculosis* complex strains from tuberculosis patients in the Littoral region of Cameroon

**DOI:** 10.1016/j.jctube.2020.100182

**Published:** 2020-09-01

**Authors:** Benjamin D. Thumamo Pokam, D. Yeboah-Manu, P.M. Teyim, P.W. Guemdjom, B. Wabo, A.B.D. Fankep, R.E. Okonu, Anne E. Asuquo

**Affiliations:** a Department of Medical Laboratory Science, University of Buea, Cameroon; b Noguchi Memorial Institute for Medical Research, University of Ghana, Legon, Accra, Ghana; c Douala Tuberculosis Reference Laboratory, Littoral Region, Cameroon; d Department of Public Health, University of Buea, Cameroon; e Department of Medical Laboratory Science, College of Medicine, University of Calabar, Calabar, Nigeria

**Keywords:** Tuberculosis, Xpert MTB/RIF, Rifampicin resistance, UgandaI sublineage, Cameroon

## Abstract

**Background:**

The re-emergence of tuberculosis (TB) worldwide, compounded by multi-drug resistance (MDR) of the causative agents constitutes a major challenge to the management of the disease. Rapid diagnosis and accurate strain identification are pivotal to the control of the disease. This pilot study investigated the genetic diversity of *Mycobacterium tuberculosis* complex (MTBC) strains from TB patients in the Littoral region of Cameroon as well as their resistance to rifampicin (RIF).

**Patients and methods:**

This was a cross sectional hospital-based study carried out between January and December 2017 and including 158 isolates from sputum smear positive individuals [105 (66.5%) males and 53 (33.5%) females]. Sputum samples were tested using Xpert MTB/RIF, followed by culture on Lowenstein–Jensen medium. Isolates were further subjected to molecular characterization using *IS6110* typing, deletion analysis and spoligotyping.

**Results:**

Thirteen (8.8%) of the 147 isolates with susceptibility results available were resistant to RIF. Drug resistance occurred in 5/50 (10%) female compared to 8/97 (8.2%) male (OR, 0.81; 0.25–2.62; p = 0.764), and there was no significant difference across the age ranges (p = 0.448). On the other hand, RIF resistance was associated (OR, 0.18, 95%CI, 0.05–0.69; p = 0.023) with previously treated patients [(4/14 (28.6%)] compared to new ones [9/133 (6.8%)]. The 150 identified lineages included among others 54 (36%) Cameroon, 18 (12%) UgandaI, 32 (21.3%) Haarlem, 17 (11.3%) Ghana, 9(6%) West African 1, 7(4.7%) Delhi/CAS, 4 (2.7%) LAM and 3 (2%) UgandaII. Of the 150 isolates, the major cluster was the Cameroon SIT 61, with 43(28.7%) isolates. Six (35.3%) of the 17 UgandaI sub-lineage were RIF resistant (OR, 9.58; 95%CI, 2.74–33.55, p = 0.001).

**Conclusion:**

The cosmopolitan Littoral region presents with a wide *Mycobacterium tuberculosis* (MTB) strains diversity and the UgandaI sub-lineage likely associated with RIF resistance. Understanding the spread of this clade through surveillance will enhance TB control in the region.

## Highlights

• The Xpert MTB/RIF provides a rapid MDR detection and management of TB patients.
• The Cameroon family is the predominant genotype in the Littoral region of Cameroon.
• The UgandaI sublineage is likely associated with RIF resistance in the study area.
• The mapping of the UgandaI sublineage is essential for MDR control in the country.

## 1 Introduction

Tuberculosis (TB) remains a major cause of illness and death worldwide, especially in Africa [1], where drug resistant TB transmission results from failure to implement proper TB control programs, including inadequate care as well as ineffective management of TB cases ranging from administration of improper regimens to failure to ensure treatment completion by patients [2], [3], [4]. Early diagnosis of TB resistance through rapid drug susceptibility testing is important for management of multidrug resistance tuberculosis (MDR-TB) [5], [6]. Conventional diagnostic methods for *Mycobacterium tuberculosis* (MTB) are slow and/or lack sensitivity [7], resulting to large proportion of TB cases as well as drug resistant TB remaining undiagnosed and leading to continuous transmission.

TB control efforts were for a long time hampered by the lack of accurate point of clinical care tests for detection of MTB and drug resistance [8], thereby delaying the initiation of TB second-line at the early stage of treatment. However, the problem has been mitigated by the development and endorsement by the WHO of the Gene Xpert® MTB/RIF assay, a rapid molecular assay which concurrently determines MTB and rifampin resistance (RR) which serves as a surrogate marker for MDR–TB [9]. Moreover, the test can be performed with minimal technical expertise and results available within 2  h, thereby allowing the early commencement of disease management [10]. The sensitivity and specificity of Xpert MTB/RIF in detecting TB have been shown to be 88% and 99% respectively, while the the sensitivity and specificity in detecting RR were 95% and 98% respectively [11].

The emergence of MDR-TB fueled by poor TB control especially in Africa as a result of limitation to funding, laboratory capacity, erratic drug supplies, qualified personnel, and facilities, [12] added to the Human Immunodeficiency Virus/ acquired immunodeficiency syndrome (HIV*/*AIDS) global pandemic [13], has contributed to the dramatic increase in the TB burden worldwide. The rpoB mutations generally found in rifampicin-resistant MTB strains are located in a region at the 507-533rd amino acid residuals (81 bp) in the MTB rpoB gene, referred to as Rifampicin-resistance-determining region (RRDR) [14]. The drug-resistant TB requires accurate diagnosis to guide therapy and interrupt transmission of resistant strains in communities [15].

Genetically diverse lineages and sublineages of MTB have evolved [16], and seven major lineages have been shown to be adapted to human sub-populations in diverse geographic settings with different variation in virulence [17], [18]. Recently, lineage 8 likely restricted to the African Great Lakes and associated MDR has been discovered [19]. Therefore, some lineages occur globally as lineages 2 and 4 probably due to high virulence, while others show geographical restriction as lineages 5 and 6, mainly restricted to West Africa. It thus appears that specific lineages have different propensities to transmit and develop drug resistance [20]. The molecular techniques have allowed the identification and tracking of individual strains of MTB [21], providing an insight into the prevalence and transmission of *Mycobacterium tuberculosis* complex (MTBC) [22]. This in turn may help improve TB control and patient management strategies [23].

IS*6110*, a 1361-bp long, belongs to a family of insertion sequences (IS) of the IS*3* category and have been utilized as targets in the identification of MTB by Polymerase Chain Reaction (PCR). The reliability, sensitivity, and specificity of PCR have been shown to be dependent on the amplification of DNA with primers specific to different target sequences in the genome [24]. It is highly conserved and has been used for the molecular epidemiological analysis of clinical isolates [25]. However, IS*6110* based diagnosis has been shown to be limited by the presence of low copy number or absence of the IS*6110* repetitive sequence. [25], [26], [27]. Spoligotyping on the other hand has some major advantages over standard IS*6110* typing, requiring minimal quantities of DNA [21] and thus can be used directly on clinical specimens without the need for prior culture. It can be valuable in countries which do not routinely culture specimens considering its ability to type isolates using small amounts of DNA. However, several samples may be required before successful typing can be performed on sputum specimens [28].

In Cameroon, TB incidence, mortality and MDR/RR-TB rate in 2018 were 186 (121–266), 31 (18–47) and 3.5 (1.7–6.0) for 100,000 population respectively [29]. Although changes in TB notification data might indicate successful TB control in the country, there are also strong indications that TB transmission is still ongoing [30]. However, there is a paucity of data on the MTBC circulating strains as well as the possible transmission of antimycobacterial drugs resistant lineages in population across the Littoral region of Cameroon. This study therefore evaluated the genetic diversity of MTBC and the associated rifampicin resistant sublineages from TB patients in the Littoral region of Cameroon which can provide a basis for TB control in the study area.

## 2 Methods and patients

### 2.1 Study design and area, specimen collection and culture

This was a prospective cross sectional hospital-based study carried out between January and December 2017 and including one hundred and fifty-eight (1 5 8) isolates obtained from TB patients attending various TB care centers across the Littoral region of Cameroon. The region is subdivided into four divisions: Wouri, Moungo, Nkam and Sanaga-Maritime with their respective capitals at Douala, Nkongsamba, Yabassi and Édéa. Douala is the economic capital of the country and is a densely populated and cosmopolitan city with foreigners as well as people coming from the other 9 regions of the country seeking for business and employment. Hence the presence of some crowded slums.

One part of an unprocessed sputum specimen from each participant was analyzed using GeneXpert MTB/RIF assay v 4.3 (Cepheid Inc., Sunnyvale, CA, USA) according to manufacturer’s instructions. Briefly, one mL of sputum samples in a 1:2 ratio of sample reagent processing solution (isopropanol and NaOH) was used [9]. The treated sample incubated at room temperature for 15 min was transferred to the cartridge and loaded into the GeneXpert instrument with subsequent fully automated processing. The other part of the sputum sample was further decontaminated using the N-acetyl L-cysteine–sodium hydroxide (NALC/NaOH) method, cultured using Lowenstein–Jensen (L-J) medium and incubated at 37 °C for 6–8 weeks. Positive slants were reconfirmed by Acid Fast Bacilli (AFB) microscopy following Ziehl-Neelsen staining technique.

### 2.2 Genotyping MTBC isolates

The preserved isolates from Cameroon in glycerol were shipped to the Bacteriology Laboratory of the Noguchi Memorial Institute for Medical Research (NMIMR) - Ghana, where approval from the Scientific Technical Committee and Institutional Review Board of the NMIMR was obtained for the genotyping of the isolates. The obtained DNA from heat-killed mycobacterial cells suspensions (95 °C for 50 min) were subjected to molecular analyses.

### 2.3 IS*6110* amplifications, deletion analyses and spoligotyping

PCR detection of the insertion sequence was carried out to confirm the MTBC as described previously [31], and the products were electrophoresed on 2% agarose gels and visualized under UV light following ethidium bromide staining.

The Large Sequence Polymorphisms (LSPs) typing assay identifying regions of difference (RD) 1, 4, 9, 12, 702, 711 was carried out on the mycobacterial DNA. Lineage-defining LSPs were detected by PCR using the primers described earlier [31] and identifying RD 1, 4, 9, 12, 702, 711 using the reactions described previously [31]. Distinct lineages within the MTBC in the study were grouped as previously defined [18], and finally, the spoligotyping was carried out following manufacturer’s instructions (Isogen Bioscience, The Netherlands) on a membrane using the 43-spacer [21]. M. tuberculosis H37Rv, and M. bovis BCG DNAs were used as parallel positive controls and distilled water as a negative control.

### 2.4 Data analysis

All the data were entered into an Excel sheet and the spoligotype patterns in a binary format were analyzed (Supplementary material I) using the SpolDB4 database/MIRU VNTR plus [32]. SPSS version 20 analyzed the association between the variables using Chi square and Fisher exact test. Values of p (two sided p-values) less than 0.05 were considered significant at 95% confidence interval.

## 3 Results

### 3.1 Rifampicin resistance by gender, age and previous treatment

Of the 158 isolates included in this study, 11 (7%) were obtained through L – J culture and not subjected to the Xpert MTB/RIF test. Of the 147 RIF susceptibility results obtained using the Xpert MTB/RIF, 13 (8.8%) were resistant and 134 (91.2%) were sensitive. One hundred and twenty-four (92.5%) of the 134 RIF sensitive were new cases. Of the 13 resistant cases, 3 (23%) occurred in relapsed patients, 1(7.7%) MDR contact, 1 (7.7%) with treatment failure and 8 (61.5%) among newly diagnosed patients. Though not significant (OR, 0.81; 95%CI, 0.25–2.62; p = 0.764), resistance occurred in 5/50 (10%) female compared to 8/97 (8.2%) male. The age groups 25 – 34 and 35 – 44 recorded [5/42 (11.9%) and [5/36 (13.9%)] resistant cases (OR,1.64; 95%CI, 0.5–5.33; p = 0.520 vs OR,2.08; 95%CI, 0.63–6.81; p = 0.308) respectively. Overall, there was no significant difference across the age ranges (p = 0.448). There was a significant association (OR, 0.18, 95%CI 0.05–0.69; p = 0.023) among previously treated patients [(4/14 (28.6%)] compared to new ones [9/133 (6.8%)] (Table 1).

**Table 1:** RIF susceptibility by gender, age and treatment status of patients.

	No. Tested	Rifampicin		Odds ratio (95% CI)	P value
		Sensitive	Resistant		
**Gender**				0.81(0.25 – 2.62)	**0.764**
Male	97	89 (91.8)	8 (8.2)		
Female	50	45 (90)	5 (10)		
**Total**	147	134	13		
**Age group (years)**					0.448
15–24	31	30 (96.8)	1 (3.2)	0.29(0.04–2.31)	0.302
25–34	42	37 (88.1)	5 (11.9)	1.64(0.5–5.33)	0.520
35–44	36	31 (86.1)	5 (13.9)	2.08(0.63–6.81)	0.308
45–54	24	22 (91.7)	2 (8.3)	0.93(0.19 –4.47)	0.642
≥ 55	14	14 (1 0 0)	0 (0)		0.367
**Total**	147	134	13		
					
**Treatment Status**				0.18(0.05–0.69)	**0.023**
New	133	124 (93.2)	9(6.8)		
Previously treated	14	10 (71.4)	4 (28.6)		
**Total**	147	134	13		

### 3.2 Genotypes distribution

**Fig. 1:**
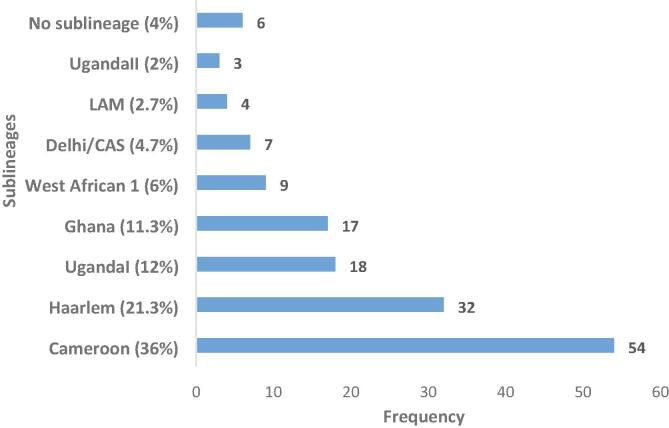
Spoligotype distribution of the isolates in the Littoral Region of Cameroon.

Eight (5.1%) of the 158 isolates were IS*6110* negative and the identified 150 isolates subdivided into 8 sublineages included 54 (36%) Cameroon, 32 (21.3%) Haarlem, 18 (12%) UgandaI, 17 (11.3%) Ghana, 9(6%) West African 1, 7(4.7%) Delhi/CAS, 4 (2.7%) LAM, 3 (2%) UgandaII and 6 (4%) with no sublineage (Fig. 1).

Table 2 shows the distribution of the 150 isolates classified into 31 identified Shared International Types (SIT). The Cameroon with SIT 61 represented the major cluster [43/150 (28.7%) isolates], followed by Haarlem SIT 50, Ghana SIT53, UgandaI SIT 52 and Delhi/CAS SIT 46 with 21(14%), 12(8%), 10(6.7%) and 6(4%) isolates respectively. Eighteen (12%) of the 150 isolates including 15 sublineages (3 Cameroon, 1 Delhi/CAS, 3 Haarlem, 2 LAM, 3 UgandaI, and 3 West African 1) did not have a SIT number, while 3 isolates with SIT numbers 1548, 847 and 852 had no identified sublineages.

**Table 2:** Distribution of genotypes, sublineages, shared international types (SITs) and spoligotypes patterns of 150 *Mycobacterium tuberculosis* complex isolates in the Littoral region of Cameroon.

### 3.3 Rifampicin resistance and sublineages association

**Fig. 2:**
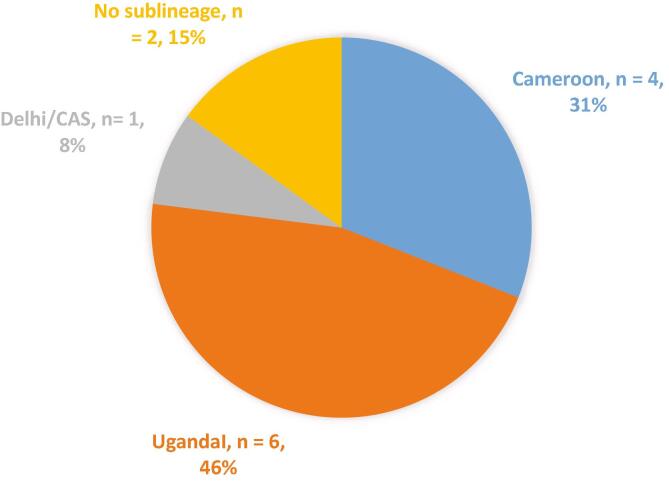
Distribution of spoligotype sublineages associated with RIF resistance.

The distribution of the sublineages according to RR is shown in Fig. 2. Six (46.2%) of 13 RIF resistant isolates were UgandaI sublineage, 4 (31%) Cameroon, 1(8%) Dehli/CAS and 2(15%) with no sublineage. The distribution of sublineages and RR in various quarters of Douala and across cities within the Littoral region of Cameroon is shown in Supplementary material II (and map in Supplementary material III), based on patients’ referral hospital location.

The six patients with RIF resistance associated with the UgandaI sublineage were reported as either treatment failure (1), relapse (2), MDR at first month of evaluation (2) or MDR contact (1), harboring three T2 [2 SIT317 and 1 SIT 52], one T1 (SIT 244), and two undefined SIT. All the terms used were based on the World Health Organisation (WHO) definition [33] as treatment failure including a patient who is sputum smear or culture positive at 5 months or later after the initiation of anti-TB treatment.

### 3.4 Rifampicin resistance association with SIT and sublineages

The association between RR with SIT and sublineages is shown in Table 3. Of the 67 isolates with SIT numbers, 9(13.4%) were RR. Four SIT [3 SIT 61 and 1 SIT 850] did not have RR results, and 3 sensitive Cameroon sublineage were without SIT number. Equally, 1 Dehli/CAS without SIT number was sensitive, 1 SIT 244 of UgandaI sublineage was without RR result and 3 (2 resistant and 1 sensitive) UgandaI sublineages had no SIT number. All the SIT of the Ghana, Haarlem, West African 1, LAM and UgandaII sublineages as well as the 3 SIT (1548, 847 and 852) with no identified sublineages exhibited no RR.

**Table 3:** Association between Rifampicin resistance with SITand sublineages.

	**No. Tested**	**Rifampicin**		**Odds ratio (95% CI)**	**P-value**
		**Sensitive n (%)**	**Resistant n (%)**		
**SIT**					
Cameroon					0.979
SIT 403	1	1 (1 0 0)	0 (0)		0.915
61	40	36 (90)	4 (10)		0.512
838	3	3 (1 0 0)	0 (0)		0.761
839	1	1 (1 0 0)	0 (0)		0.915
844	1	1 (1 0 0)	0 (0)		0.915
850	1	1 (1 0 0)	0 (0)		0.915
**Total**	**47**	**43 (91.5)**	**4 (8.5)**		
UgandaI					0.02
SIT 244	1	0 (0)	1 (1 0 0)		0.286
3	1	1 (1 0 0)	0 (0)		0.714
317	2	0 (0)	2 (1 0 0)		0.659
52	10	9 (90)	1 (10)	0.04(0.0–0.79)	0.04
**Total**	**14**	**10 (71.4)**	**4 (28.6)**		
Delhi/CAS					
SIT 46	6	5 (83.3)	1 (16.7)		
**Total**	**67**	**58 (86.6)**	**9 (13.4)**		
**Sublineages**					0.005
UgandaI	17	11 (64.7)	6 (35.3)	9.58(2.74–33.55)	0.001
Cameroon	50	46 (92)	4 (8)	0.85(0.25–2.91)	0.53
Delhi/CAS	7	6 (85.7)	1 (14.3)	1.78(0.2–16.02)	0.484
UgandaII	3	3 (1 0 0)	0 (0)		0.756
LAM	3	3 (1 0 0)	0 (0)		0.756
West African 1	9	9 (1 0 0)	0 (0)		0.606
Haarlem	30	30 (1 0 0)	0 (0)		0.071
Ghana	16	16 (1 0 0)	0 (0)		0.3617
No sublineage	12	10 (83.3)	2 (16.7)	2.25(0.44–11.61)	0.601
**Total**	**147**	**134 (91.2)**	**13 (8.8)**		

Among the Cameroon sublineage, 4/40 (10%) SIT 61 (p = 0.488) were RIF resistant. One (100%) SIT 244, 2 (100%) SIT 317 and 1/10 (10%) SIT 52 (OR, 0.04; 95%CI, 0.0–0.79; p = 0.04) within the UgandaI sublineage (p = 0.02) were RIF resistant. One (16.7%) of the 6 Dehli/CAS was RIF resistant

Of the 147 sensitivity tests performed, 2 (1.4%) resistant and 10 (68%) sensitive strains had no sublineage. All the 3 (100%) UgandaII, 3 (100%) LAM, 9 (100%) West African 1, 30 (100%) Haarlem and 16 (100%) Ghana sublineages were sensitive to RIF. The number exhibiting RR included 6/17 (35.3%) UgandaI (OR, 9.58; 95%CI, 2.74–33.55, p = 0.001), 4/50 (8%) Cameroon (OR, 085, 95%CI, 0.25–2.91; p = 0.53) and 1/7(14.3%) Dehli/CAS (OR, 1.78; 95%CI, 0.2–16.02; p = 0.484) sublineages.

## 4 Discussion

This study carried out in the Littoral region of Cameroon has shown a RR of 8.8%, similar to a research carried out in South Africa [34] but higher than a previous one in Zambia [35]. RR is regarded as a proxy for MDR-TB. Currently available molecular assays have the advantage of rapidly detecting resistant strains of MTB, but the GeneXpert does not detect isoniazid (INH) resistance [34]. Nevertheless, some evaluation studies have shown that using Xpert MTB/RIF instead of smear microscopy as currently practiced in several developing countries can tremendously improve TB detection [36], [37]. The large scale implementation and widespread extension policy of the use of Xpert MTB/RIF in Cameroon, is an asset in the rapid diagnosis of TB in the country, especially as culture is not carried out routinely due to limited facilities. In 2018, Noeske and coworkers [38] reported GeneXpert based RR of 1.6% among new cases of TB in 10 regions of Cameroon with regional variations ranging from 0 to 3.3%. The higher prevalence in our study could be explained by the fact that our data was generated from both retreatment and newly diagnosed TB patients. The diagnosis of extensively drug-resistant (XDR) TB using the Xpert MTB/XDR assay for INH and second-line drug resistance currently under clinical evaluation trial, will be an added value in the rapid detection of XDR-TB strains [39], which magnitude is largely unknown in the country.

RIF resistance in this study was not associated with gender.as shown in similar studies [35], [40]. It has been shown in general that there was no evidence of either sex being more at risk of MDR/RR-TB in 81% (86/106) of countries using sex-disaggregated data of TB patients reported to WHO [41]. In an earlier report, Coovadia *et al*. [34] showed that males had an increased odd of being RIF mono-resistant as compared to females, and that RIF resistance is more likely to occur in the 25–29 years’ age category. It has been shown in a neighboring country that significantly higher proportion of TB patients aged above 45 years had RR compared with patients below 45 years [42], in contrast to our study which found no age related association.

This study has shown that previously treated patients were significantly infected with RIF resistant strains unlike a study carried out in Ethiopia who found no association compared to new patients [43], but was in line with a neighboring country - Nigeria where a significant proportion of RR-TB was found among previously treated TB patients [42]. Several studies have linked history of previous TB treatment as a strong risk factor for MDR-TB [44], [45], calling for a surveillance strategy to be implemented in order to control the dissemination of MDR strains by patients on retreatment. Improvement of treatment adherence as well as laboratory capacities and introduction of molecular diagnostic tools detecting the various TB genotypes will be required for an efficient control not only of MDR dissemination, but equally MDR associated lineages to prevent the likely spread of MDR-RR strains in the country.

The predominant sublineage in our study was the Cameroon family, which has earlier been shown not only to be prevalent in the country, but largely implicated in different pocket of TB transmission [46]. The spoligotype 61 especially which lacks spacers 23, 24, and 25 in the direct repeat (DR) region represented about 29% of all the isolates (and about 80% of the Cameroon family in this study) has been shown to be widely prevalent Cameroon [46], [47], [48], Nigeria [31], [49], Chad [50], and within the west African region [51], [52].

The Cameroon family (36% in this study) seems well established since its designation and description in the west region of Cameroon (more than 40%) nearly two decades ago [48]. A similar study as ours carried out recently in the Littoral region specifically in Douala the city capital, recorded 54% of the Cameroon family with 51% of SIT61 [53], thereby buttressing the clustering of pulmonary TB in this city [54].

The other families in this study such as the H (lineage H1 and H3), T (lineage T1, T2 and T5) and U with lineage U and U (Likely H) have been previously described in the country [46], [55], as well as in Douala [53]. The latter authors recorded in line with our study that, the Haarlem sublineage, especially SIT 50 was the second most predominant one encountered. Koro Koro and colleagues [46] in their study in the Adamaoua region of Cameroon reported the ubiquitous T family and in addition, noted the significant presence of the H1 family, suggesting an adaptation of these strains to the local population following their introduction through migration. These observations have been noted elsewhere [49], [56]. In our study, *M. africanum* (MAF) represented 6% with 2% belonging to SIT 101. This corroborates with the findings of a previous study carried in the same study area which identified 2.74% MAF1 represented by AFRI_2 lineages [53]. The contribution of MAF to TB disease has substantially decreased from 56% over the past four decades, accounting for only 9% of cases about 20 years ago in the country [48]. In neighboring Nigeria, it accounted for 12% a decade ago [49].

This study has shown the UgandaI sublineage as well as SIT 52 within the UgandaI sublineage likely associated with RIF resistance. This is contrary to a study carried out in Uganda which showed through a cluster analysis no significant association between drug resistance and lineages, especially among the T2 family [57]. Previous studies in Cameroon have shown no statistical link between drug resistance and MTBC genotypic families [46], [47]. It could have been hypothesized instead an association of drug resistance with the most prevalent Cameroon sublineage (especially SIT61) present in the country. Equally, this lack of association has been demonstrated recently in a neighboring country [31]. The Uganda genotype of MTB has been shown not only to be the prevalent (up to 70% of isolates) cause of PTB in Uganda [58], but equally associated with extrapulmonary TB [59]. Kigozi *et al*. [60] in a study assessing RR in MTB isolates from Uganda found that lineage 4/sub-lineage Uganda accounted for 36% of the rifampicin-resistant isolates with 24% being UgandaII and 11% UgandaI. This is lower compared to our findings, where all the six patients with RIF resistance associated with the UgandaI sublineage had pulmonary TB. Also in their study, patients were either on treatment failure, relapse, MDR at first month of evaluation or MDR contact. Noeske *et al*. [61] in a non-molecular based study, recorded 12% MDR strains in the Littoral region with low positive treatment outcome rates in retreatment patients with MDR-TB. Although TB drug resistance has been linked both to the quality of control programs as well as socioeconomic status, the intrinsic factors prompting its emergence and expansion remain unclear [62], [63]. However, there is evidence of genotypic linkage of MTB strains driving the epidemiology of drug resistant TB isolated from patients in different geographical region and suggesting and adaptation of various lineages to particular genetic, cultural or environmental characteristics of the host [64]. This might be an evolutionary trend which will require further careful investigation to measure the real impact of this association in the Littoral region of Cameroon. The emergence and spread of this drug resistant MTB lineage originally absent in this region as shown by a previous study [53], could be associated with immigration, clinical and demographic factors, as well as evolution of MTB strains. Understanding the mechanisms shaping transmission and regardless of whether the patients acquired the infection elsewhere or from their current locality or from a reactivated disease contracted in their native country can provide an insight into the potential approaches for TB control in this setting [65], [66]. Although a hypothesis suggesting that lineages not previously described in a defined population earlier could be introduced by immigrants, paucity of information and data on various drug resistant genotypes especially in Africa makes this assumption difficult to prove. The introduction of new MTB strains that more transmissible and virulent and more prone to develop drug resistance has been associated with migration, (especially movement of population to bigger cosmopolitan cities in search of better health care facilities as well as employment opportunities) and can drive the current changing TB situation [67]. In the current context of globalization and population movement, this is a particular challenge in the control of drug resistant TB strains dissemination. Douala our study area, being not only the economic capital of the country with attendant high population overcrowded slums, is additionally the main entry point in Cameroon with a seaport and the busiest airport of the country bringing in foreigners. This can thus further explain the introduction of the UgandaI sublineage in this setting.

The circulating MTBC strains surveillance in a locality is important for understanding TB epidemiology. MTB strain identification can contribute to the better control of the disease [68]. Newer strategies including specific active surveillance especially of relapsed and retreatment patient should be elaborated for efficient control of the surge of MDR and associated lineages in the region. The prevalence of the UgandaI genotype likely associated with RR in the Littoral region of Cameroon might suggests a recent introduction as well as result of poor treatment adherence considering that all the patients involved were either on retreatment or MDR contacts. Furthermore, this genotype has not been previously associated with drug resistance in the country. Hence, its adaptation in a somewhat virgin population might explain its virulence and development of resistance.

### 4.1 Study limitations

The small sample size utilized in this study may not permit definitive conclusions and so a larger scale study in the region is necessary to understand the epidemiological trends and transmission pattern of the UgandaI sublineage. Thus, further evaluation to determine the real impact and magnitude of the transmission of the UgandaI sublineage associated with RR not only in the region but equally across the country will be necessary.

The hospital referral centers were used to map the location of the patients, rather than their actual residence. This was based on the assumption that patient first seek care to the closest hospital center which served here as a referral site, and as such, it is instead the referring hospitals geographic position that was used to determine the clusters. The exact residence coordinate and history of patients would have been useful for understanding the transmission and clustering patterns of the sublineages found in this study.

## 5 Conclusion

The Xpert MTB/RIF does provide a rapid surrogate MDR detection and thus a timely management of TB patients, preventing the associated increased morbidity and mortality in the study area. The cosmopolitan Littoral region presents with a wide MTB strains diversity, with the predominant being the Cameroon family and the UgandaI sub-lineage likely associated with RIF resistance. Clear mapping and understanding the current trend of dissemination of the UgandaI sublineage is essential for the control and development of the drug resistance associated with this clade, which appears to constitute a flash point in the study area, country and sub-region.

## Ethical statement

This research was approved by the Scientific Technical committee and Institutional Review Board of the Noguchi Memorial Institute for Medical Research (NMIMR), University of Ghana, Legon, Accra – Ghana [FWA 00001824; IRB 00001276; NMIMR-IRB CPN 007/16-17; IORG 0000908].

## Declaration of Competing Interest

The authors declare that they have no known competing financial interests or personal relationships that could have appeared to influence the work reported in this paper.
